# Dilated Cardiomyopathy in Children: Early Detection and Treatment

**DOI:** 10.7759/cureus.31111

**Published:** 2022-11-04

**Authors:** Amrita Mallavarapu, Amar Taksande

**Affiliations:** 1 Department of Paediatrics, Jawaharlal Nehru Medical College, Datta Meghe Institute of Medical Sciences, Wardha, IND; 2 Department of Pediatrics, Jawaharlal Nehru Medical College, Datta Meghe Institute of Medical Sciences, Wardha, IND; 3 Pediatrics, Jawaharlal Nehru Medical College, Wardha, IND

**Keywords:** heart transplantation, arrhythmia, heart failure, dysfunction, left ventricle, dilated ventricle, cardiomyopathy

## Abstract

Cardiomyopathy is segregated into primary and secondary categories, leading to different phenotypes, including dilated, hypertrophic, and restrictive patterns. Dilated cardiomyopathy (DCM) is a mixed bag of heart diseases with the unique features of cardiac dilatation and subnormal to poor myocardial contractility. Dilated cardiomyopathy in the pediatric age group is generally characterized by unobstructed, dilated, and contracting left ventricular chamber defects and is associated with heart failure. Other causes include genetic juvenile-onset cardiomyopathy, drug-induced cardiomyopathy, stress-induced cardiomyopathy, hemochromatosis, endocrine causes (thyroid disorder and pheochromocytoma), autoimmune diseases, and nutritional deficiencies (selenium and thiamine). It is characterized by thinning of the left ventricle, a dilated left ventricle or biventricular dilatation, left ventricular systolic dysfunction, left ventricular diastolic dysfunction, global hypokinesia, and cardiomegaly (seen on a chest X-ray). Decreased cardiac output leads to fatigue, cachexia, narrow pulse pressure, dicrotic pulse/hypokinetic pulse, dyspnea, cool extremities, decreased blood supply to the brain (cognitive dysfunction), and reduced blood supply to the kidney (renal failure). Left ventricular diastolic dysfunction can result in dyspnea, orthopnea, and paroxysmal nocturnal dyspnea. Cardiomyopathy can also occur with or without left ventricular dysfunction, as in left ventricular non-compaction cardiomyopathy (LVNC), which is a rare heart disease occurring due to two (likely) pathogenic nonsense mutations: DSG2-p.S363X and TBX20-p.D278X. The study of familial forms of LVNC is helpful for risk prediction and genetic counseling of relatives. Right ventricular chamber failure can be identified on the electrocardiogram as sinus tachycardia and non-specific ST/T changes. The complications include arrhythmia (atrial fibrillation) and thromboembolism (left ventricular mural thrombosis). It is of utmost importance in the field of heart transplantation as the definitive treatment of the disease. Heart transplantation is now an acceptable treatment option for patients with dilated cardiomyopathy. This short review highlights different methods for the detection and diagnosis of dilated cardiomyopathy and the treatment modalities available for the same.

## Introduction and background

Cardiomyopathies are diseases of the heart that affect the systole, diastole, or both phases of the cardiac cycle. Cardiomyopathy can be divided into primary (primary is also divided into genetic, mixed, or acquired types) and secondary categories, which has ultimately led to varied phenotypes that include dilated, hypertrophic, and restrictive patterns. Based on specific morphological and functional phenotypes, cardiomyopathies can be clinically classified into dilated cardiomyopathy, hypertrophic cardiomyopathy, restrictive cardiomyopathy, and arrhythmogenic right ventricular dysplasia, as shown in Table 1.

**Table 1:** Types of cardiomyopathies Cardiomyopathies can be classified into four different groups according to different morphological and functional criteria [1].

Types of dilated cardiomyopathies
Dilated cardiomyopathy
Hypertrophic cardiomyopathy
Restrictive cardiomyopathy
Arrhythmogenic right ventricular dysplasia

Most patients have been found to have "pure" forms of the above, which completely adhere to the diagnostic criteria. However, some patients have an overlap of symptoms with mixed conditions of cardiomyopathies [1]. In hypertrophic cardiomyopathy (HCM), there is excessive ventricular chamber hypertrophy with smaller than usual cavity sizes of the ventricles. The contraction ability of the ventricle is seen to be enhanced, but the filling of the ventricles is impaired secondary to abnormalities in relaxation. Dilated cardiomyopathy (DCM) denotes a reduced contraction function of the ventricle along with dilatation of the ventricle. Endocardial fibroelastosis (a condition usually seen in infancy) and cardiomyopathy secondary to doxorubicin (seen explicitly in children receiving chemotherapy for malignant conditions) have clinical signs and symptoms related to that dilated heart disease. RCM is characterized by the decreased filling function of the ventricles during the diastolic phase (usually seen in infiltrative conditions). The contraction function of the ventricular chambers may be expected, but a marked dilatation of both atria is noticed.

DCM, a myocardial disorder, is recognized as a left ventricle that is dilated and is a frequently occurring type of cardiomyopathy. It is one of the critical diseases treated by cardiac transplantation in both the pediatric and adult age groups. It is of utmost importance in the field of heart transplantation as the definitive treatment of the disease. The utilization of heart transplantation has led to improved survival after the diagnosis of pediatric DCM. Most of the deaths occur within one year of presentation, which suggests that most of the patients are not diagnosed until they have an end-stage disease that is not effectively palliated with the medical therapies used to treat them [2]. If not diagnosed early in life, this disease will become a global burden all over the world. As shown in a study conducted by Tsirka AE [2], the actual survival of the children from the time of diagnosis was 90% at one year (82 of 91) and 83% (76 of 91) at five years, which highlights the importance of early diagnosis of DCM so that appropriate support and treatment could be provided. At times, right ventricular malfunction is also seen in addition to the usually observed left ventricular dilatation and malfunction.

This review article highlights the various methods for the detection and treatment of dilated cardiomyopathy in children.

## Review

Epidemiology

DCM is usually due to left ventricular (LV) dilatation. The patient might additionally have right ventricular dilatation, but it is of no great importance in diagnosing the condition as it can be present in several conditions, including pulmonary valve stenosis. pulmonary arterial hypertension (PAH) or an atrial septal defect (ASD). The prevalence of the disease (DCM) is seen in all age groups, races, and ethnicities. In children, the incidence is around 0.57 per 100,000 population and is usually seen more frequently in boys than girls, with incidences of 0.66 per 100,000 population and 0.47 per 100,000 population, respectively [3]. Arola et al. reported an incidence of DCM of 0.34 cases per one lakh children per year and a prevalence of 2.6 points per one lakh children in Finland, among a racially uniform group of people [4]. In about 66% of the cases, the cause is idiopathic. At times, the disease is an accidental finding and is severely underdiagnosed [4-12]. A considerable number of cases happen to be present in children younger than one year of age: 3.8 points per 1 lakh cases every year [13,14].

Etiology

**Figure 1:**
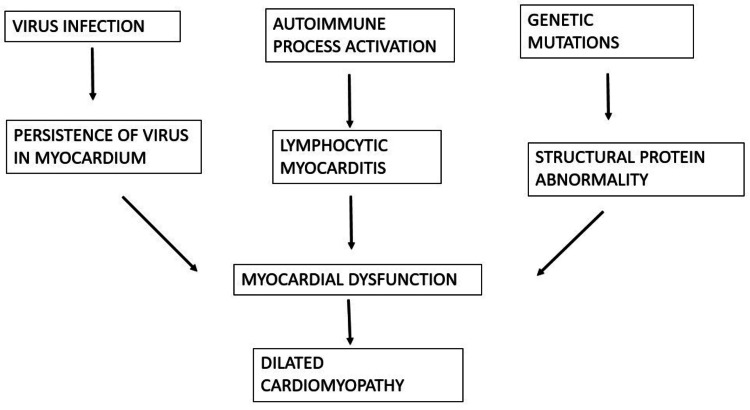
Etiological factors causing dilated cardiomyopathy Myocardial dysfunction can be caused by the persistence of a virus in the myocardium after a viral infection, an autoimmune process leading to lymphocytic myocarditis, or genetic mutations leading to structural protein abnormalities (which can further cause electrical abnormalities), leading to myocardial dysfunction that ultimately causes dilated cardiomyopathy [20,23].

Cardiomyopathy can be primary or secondary, depending on the etiology. Primary cardiomyopathies can have a genetic cause, or they can also be acquired. Secondary cardiomyopathies are secondary to systemic or multiorgan disease causing myocardial damage. At times, it is diagnosed as an incidental finding during the evaluation of some other associated cardiac disease, usually by two-dimensional echocardiography. Cardiomyopathies are a frequent cause of inpatient admission in hospitals and are the most typical cause of the need for heart transplantation procedures in patients of all age groups [15-17]. DCM in the pediatric age group is generally recognized by unobstructed, dilated, and contracting left ventricle defects and is associated with heart failure [18-20]. The usual causes of DCM include inflammation of the myocardium and neuromuscular disease. Duchen or Becker muscular dystrophies, in which a definite viral association concerning these neuromuscular disorders was also found in very few cases. The evidence of viral myocarditis is common in children with DCM parvovirus B19, influenza, Ebstein-Barr virus, human immunodeficiency virus, coxsackie, herpes, and adenoviruses, which have all been found. Pediatric DCM is also observed to manifest after exposure to certain toxins, such as anthracyclines, during chemotherapy [20]. The causes are usually indeterminate. There are fewer studies on the pathophysiology of this condition in the pediatric age group because of the comparatively small patient population and the unique challenges in separating other types of heart diseases associated with dilation of the ventricle chambers, namely endocardial fibroelastosis or myocarditis [20-23]. A significant chunk of the cases of genetic DCM was observed to be inherited in autosomal dominant patterns with variable expressivity and penetrance. There were also certain specific forms inherited in autosomal recessive, x-linked recessive variants, and mitochondrial inheritance types, which can account for 20%-48% of cases. It is associated with conditions like mitral regurgitation and ventricular arrhythmias like atrioventricular nodal blocks, supraventricular tachycardias, and atrial fibrillation. Despite a deep search for the etiologies of DCM using recent advances, most of the cases have been designated as idiopathic without the presence of an etiology. With the available categorization systems, cardiomyopathies in children can also be divided into (a) morphology types (DCM, HCM, and restrictive cardiomyopathy (RCM)); (b) etiological types (primary or secondary); and (c) pathological types (ischemic or nonischemic). Different aetiological factors have been highlighted in Figure 1 below.

 

On pathologic examination

Grossly dilating all four chambers of the heart can be identified as a flabby, hypocontracting heart. On histopathology, a) hypertrophied muscle fibers can be identified, and b) ninja star nuclei (irregular and hyperchromatic), are seen due to a titin gene mutation. c) box-car nuclei are seen in hypertensive and atherosclerotic disease; d) Tokotsubo cardiomyopathy; and e) arrhythmogenic cardiomyopathy. Tokotsubo cardiomyopathy, a type of DCM found in situations of extreme emotional stress (broken heart syndrome), is characterized by selective left ventricle ballooning in which the neck of the left ventricle becomes narrow with a round bottom, resembling the Japanese octopus trap (Takotsubo). This cardiomyopathy is more commonly found (mostly) in elderly females than in males. Troponin-one is elevated, and ST-segment elevation can be identified on the electrocardiograph (ECG). Arrhythmogenic cardiomyopathy, an autosomal dominant disorder, is generally characterized by right ventricular dysfunction caused by mutations of plakoglobin or desmin. Naxos syndrome is arrhythmogenic cardiomyopathy characterized by hyperkeratosis of the palms and soles.

Clinical features

Patients can present with symptoms of overt heart failure, including sweating, breathlessness, orthopnea, and decreased exercise tolerance, to name a few. Young children usually show a reduced appetite and cachexia. Sinus tachycardia, jugular venous pressure distension, pallor, and hepatomegaly are some of the clinical signs that can be seen. The late signs include peripheral edema and abdominal distension. At times, a murmur of mitral regurgitation can be heard. Despite this, clinical signs and symptoms observed in most children with heart disease include discomfort, weakness, breathlessness after activity, and pain in the chest. Other commonly found signs and symptoms common to most children with cardiomyopathies include arrhythmias (which can be an increased or decreased heart rate), inflammation of the innermost layer of the heart wall known as endocarditis, and congestive heart failure or abrupt death due to cardiac failure. The hallmarks of the disease include a dilated left ventricle, biventricular dilatation, left ventricular (LV) systolic dysfunction or thinning of the LV, LV diastolic dysfunction, global hypokinesia, and cardiomegaly. Decreased cardiac output leads to fatigue, cachexia, narrow pulse pressure, dicrotic pulse/hypokinetic pulse, dyspnea, cool extremities, reduced blood supply to the brain (cognitive dysfunction), and decreased blood supply to the kidney (renal failure). LV diastolic dysfunction leads to dyspnea and orthopnea. Right ventricular failure can be identified on the ECG as sinus tachycardia and non-specific ST/T changes. The complications of which include arrhythmias (atrial fibrillation) and thromboembolism (LV mural thrombosis), which can eventually lead to sudden cardiac failure and death. Cardiomyopathy can therefore be considered a differential diagnosis for sudden cardiac failure and death in children in the pediatric age group.

Diagnosis

The diagnosis of the condition is made through a multifold approach that includes asking about the family history to take into consideration the genetic predisposition and environmental factors, the clinical history, the physical examination findings, blood investigations, and various modalities of radiography and echocardiography (ECG).

The genetic basis of dilated cardiomyopathy highlights the importance of screening at-risk family members. The basic evaluation of a family in which DCM is present should include a detailed family history with a minimum of three generations and an initial clinical screening of first-degree relatives [16,23]. A study conducted by Michels VV on the relatives of 59 index patients with DCM, in which a total of 315 patients were examined, found that at least one in five of the patients in this study had DCM, which implies the importance of family screening [24].

The chest X-ray usually shows hypertrophy of the heart chambers and signs of pulmonary venous congestion. More commonly, notching at the ribs can be seen in Takaya's arteritis than in coarctation in an elder child suspected of heart disease. The electrocardiogram most commonly shows atrial fibrillation or sinus tachycardia, arrhythmias (usually ventricular), hypertrophy of the left atrium, and sometimes intraventricular conduction defects and low current. In cases of dilated or congestive cardiomyopathy, the left bundle-branch block (LBBB) is also seen with a deviation toward the right axis, which is not a common finding together. Electrocardiography can be used to see the presence of sinus tachycardia, conduction blocks, and left ventricular enlargement changes.

Echocardiographic evaluation of the presence of dysfunction in the ventricles is considered the best method for the assessment and diagnosis of various types of heart diseases in pediatric age groups and adolescents. Even though two-dimensional morphological echo and ejection fraction are the critical elements for phenotype characterization, in echo, morphologically, we can identify biventricular dilatation as an increase in left ventricular end-diastolic dimension (LVDD) and left ventricular end-systolic size (LSVD), enlargement of the atrium in relation to the ventricular enlargement, decreased LV contractility, and an apical thrombus. It also shows a reduced ejection fraction along with dilatation of the left ventricle with regular or thin walls.

Cardiac magnetic resonance imaging (MRI) can be done to understand the extent of the dilatation of the ventricular chambers, the morphology, the fibrosis, the pumping capacity of the heart, and where irregular heartbeats originate, which is essential in determining the type and severity of cardiomyopathy and to rule out arrhythmogenic right ventricular cardiomyopathy (ARVC). On cardiac MRI, the left ventricular dimensions and functions, including strain measurements, can be appropriately measured. Gadolinium, which is used as a contrast agent, is helpful in evaluating fibrosis and is used to provide information about the quality of the myocardial tissue. In dilated cardiomyopathy, the amount of fibrosis and delay in the enhancement of gadolinium contrast are predictors of mortality and also predict the necessity of future hospitalization.

A few blood investigations can be used as biomarkers for diagnosing genomic mutations, which is helpful in the management and also as a prognostic indicator. They include ventricular natriuretic peptide and N-terminal pro-brain natriuretic peptide (NT-BNP). The levels of BNP have markedly increased [25]. Endomyocardial biopsy may be of use in a select number of patients, for example, those with suspected cardiac hemochromatosis and other infiltrative or malignant diseases-but in general, it should be confined to carefully conducted clinical trials [26]. On histological and microscopic examination, biopsies of the endomyocardial specimens, along with the complementary role of electron microscopy, will provide the physician with valuable information for a confirmatory diagnosis of pediatric cardiomyopathies. As stated by G. Takemura et al., "a case of cardiac sarcoidosis patient" [27], the biopsy specimens are too small for detection of the specific sarcoid lesions, which are usually randomly distributed and scattered throughout. In this case, we could not specify sarcoidosis by light microscopic examination of the first biopsy specimens because only nonspecific inflammatory findings were observed. However, the electron microscopic analysis clearly revealed the presence of some epithelioid cells. This suggests that, when small tissue specimens are used, such as endomyocardial biopsy specimens, electron microscopy may sometimes be superior to light microscopy for the detection of epithelioid cells. Transmission electron microscopy (TEM) has been utilized to document ultrastructural changes in the heart in cases of pathological remodeling, such as hypertrophy, hypertension, cardiomyopathy, and ultimately progression to heart failure (HF). TEM is a powerful tool for not only visualizing the cardiac ultrastructure at a higher degree of resolution and magnification in comparison to traditional microscopy and imaging techniques but also for shedding significant light on the contribution of mitochondrial-derived signaling processes to dysregulation of the myocardium during health and disease [28].

The assessment of cardiac anomalies in systolic function (excitability and ability to contract), diastolic function (relaxation and compliance), and myocardial growth (hypertrophy and atrophy) is the hallmark of the differential diagnosis of pediatric cardiomyopathies. Anomalous origin of the left coronary artery from the pulmonary trunk (ALCAPA) must be ruled out as it is a rare cause of left ventricular dysfunction in infants. Its recognition is important because of the superior results of surgery as opposed to medical management. Its recognition can be done using cross-sectional echocardiography followed by confirmatory contrast cine arteriography, which remains the definitive investigation in these patients [29].

Treatment 

The goals of medical management are to reduce the symptoms of CHF and thereby stop the progression of the disease. Although medical therapy remains a mainstay in treating patients, it is not the definitive management. The primary aim is to control symptoms and prevent disease progression and complications, and it also improves the quality of life of symptomatic DCM patients [30]. The drugs used in medical management include angiotensin-converting enzyme (ACE) inhibitors and beta-blocker classes of drugs, with or without diuretics [31]. The presence of heart failure indicates the use of diuretics. Vasodilators like nitroglycerine and nesiritide can be used. The safest drug that can be used in the pediatric age group with the fewest side effects is carvedilol, a beta-adrenergic blocker with additional vasodilating action. Ionotropic support is indicated in the presence of hypotension in such cases.

Dopamine and dobutamine should be administered intravenously to partially reverse chronic congestive heart failure and additionally improve heart function temporarily; these are the sympathomimetics belonging to the ionotropic group of drugs. Still, excessive use can lead to arrhythmia and myocardial irritability. Milrinone is the preferred agent in the pediatric population. It has been observed that in patients with poor myocardial excitation and contraction along with symptomatic arrhythmias, amiodarone can be used, a class III agent widening the action potential belonging to the antiarrhythmic group of drugs. There are many nutrients benefiting patients with cardiovascular disease that might reverse myocardial dysfunction, including L-arginine, propionyl-L-carnitine, and coenzyme Q10 [32,33]. Recombinant human growth hormone resulted in improvement of the left ventricular ejection fraction when administered as subcutaneous injections or conventional therapy in children with dilated cardiomyopathy. But also, growth hormone therapy was associated with increased somatic growth [34-36]. Cardiac resynchronization therapies are used in pediatric age groups in the presence of advanced heart failure and conduction delay conditions. Cardiac transplantation remains the mainstay and is reserved for advanced and extreme cases. The outcome predictors in children have been attributed to several factors, but the disease course has often been termed unpredictable. In the absence of a heart donor or patients awaiting a transplant and with advanced disease, ventricular-assist devices have shown improvement in the quality of life of the patients [37]. There are also various post-transplant complications that can occur in children, including late renal dysfunction, with a few progressing to dialysis or a renal transplant. Infections are prevalent during the whole post-transplant period, with bacterial infections being the most common during the first month after transplantation and causing deaths.

Prognosis

Most infants with DCM die within the first year of their lives as soon as they are diagnosed, with survival rates at one and five years of 79% and 61%, respectively [38-45]. Most children die of heart failure in early childhood, and the rest of the children whose ventricular function did not fully recover can fail due to arrhythmias.

## Conclusions

Dilated cardiomyopathy remains an enigma. Despite the intensive search for etiology, the underlying cause of most cases remains unknown. Despite the extensive quest for an etiology, most cases have an unknown reason. Multiple causes are probably present, with many of them leading to clinically identical cardiac myopathies. Because patients' outcomes range from complete recovery to sudden death, characteristics that predict results should be identified so that treatment solutions for a single patient can be selected appropriately. Furthermore, recognizing patients with the worst outcomes should aid in determining the best time for a heart transplant. Despite recent developments in medicine and surgical procedures, various novel investigations and treatment approaches have been offered with little success. In treating the condition, stem cell therapy has shown some promise. Although they were not so successful, palliative surgical techniques were also tried as a bridge to heart transplantation. The future of disease therapy remains an intriguing topic of study, but for the time being, traditional treatment methods continue to be prioritized. Early detection and treatment of the condition may be aided by screening echocardiography.
